# Supplementary material for: Stacking Model‐Based Classifiers for Dealing With Multiple Sets of Noisy Labels
Source: Biom J. 2025 Mar 12;67(2):e70042. doi: 10.1002/bimj.70042 (PMC11898607; doi:10.1002/bimj.70042)
Supplement: Supplementary file 1 — Supporting Information [file BIMJ-67-e70042-s001.zip › density-based-ensemble-model-main/competitors_real_data/netlab-master/netlab-master/help/netopt.htm]

Netlab Reference Manual netopt


# netopt

## Purpose

Optimize the weights in a network model.

## Synopsis

```
[net, options] = netopt(net, options, x, t, alg)
[net, options, varargout] = netopt(net, options, x, t, alg)
```

## Description

`netopt` is a helper function which facilitates the training of
networks using the general purpose optimizers as well as sampling from the
posterior distribution of parameters using general purpose Markov chain
Monte Carlo sampling algorithms. It can be used with any function that
searches in parameter space using error and gradient functions.

`[net, options] = netopt(net, options, x, t, alg)` takes a network
data structure `net`, together with a vector `options` of
parameters governing the behaviour of the optimization algorithm, a
matrix `x` of input vectors and a matrix `t` of target
vectors, and returns the trained network as well as an updated
`options` vector. The string `alg` determines which optimization
algorithm (`conjgrad`, `quasinew`, `scg`, etc.) or Monte
Carlo algorithm (such as `hmc`) will be used.

`[net, options, varargout] = netopt(net, options, x, t, alg)`
also returns any additional return values from the optimisation algorithm.

## Examples

Suppose we create a 4-input, 3 hidden unit, 2-output feed-forward
network using `net = mlp(4, 3, 2, 'linear')`. We can then train
the network with the scaled conjugate gradient algorithm by using
`net = netopt(net, options, x, t, 'scg')` where `x` and
`t` are the input and target data matrices respectively, and the
options vector is set appropriately for `scg`.

If we also wish to plot the learning curve, we can use the additional
return value `errlog` given by `scg`:

```
[net, options, errlog] = netopt(net, options, x, t, 'scg');
```

## See Also

`netgrad`, `bfgs`, `conjgrad`, `graddesc`, `hmc`, `scg`

---

**Pages:**
Index


---

Copyright (c) Ian T Nabney (1996-9)
